# Experiences and attitudes related to newborn feeding in central Uganda: A qualitative study

**DOI:** 10.1371/journal.pone.0274010

**Published:** 2022-10-19

**Authors:** Andrew Sewannonda, Alvaro Medel-Herrero, Victoria Nankabirwa, Valerie J. Flaherman

**Affiliations:** 1 Department of Epidemiology and Biostatistics, School of Public Health, College of Health Sciences, Makerere University, Kampala, Uganda; 2 Department of Pediatrics, School of Medicine, University of California, San Francisco, San Francisco, CA, United States of America; Flinders University, AUSTRALIA

## Abstract

**Objective:**

Adequate infant nutrition is a critical cornerstone of population health, yet adherence to recommended breastfeeding practices is low in many countries in sub-Saharan Africa, including Uganda. This study aims to describe local attitudes, experiences and beliefs related to nutrition in early infancy in Central Uganda

**Design:**

We conducted 5 focus group discussions and 12 key informant interviews to gather information on local attitudes, experiences and beliefs related to feeding in early infancy.

**Setting:**

Urban areas of Central Uganda.

**Participants:**

Parents and healthcare and public health professionals.

**Results:**

Participants reported numerous concerns related to infant health including inadequate infant weight, premature birth, diarrhea, fever, gastrointestinal infection and malnutrition. Awareness of the infant health benefits of exclusive breastfeeding was prevalent but experienced as in balance with maternal factors that might lead to supplementation, including employment demands, physical appearance, pain, poverty and maternal health and malnutrition. Breastfeeding was highly valued, but use of unsafe breast milk supplements was common, including cow’s milk, black tea, glucose water, fruit juice, millet, maize, rice, potatoes, soy, sorghum, egg yolk, fish and ghee. Expression of breast milk was viewed as not consonant with local culture.

**Conclusions:**

Participants were aware of the benefits of exclusive breastfeeding but described multiple barriers to achieving it. Supplementation with unsafe breastmilk supplements was considered to be more culturally consonant than milk expression and was reported to be the only affordable potential breast milk substitute for many families.

## Introduction

Malnutrition during early infancy has substantial consequences for global health, increasing morbidity and mortality throughout childhood and causing delays in cognitive development that may have lifelong significance [1]. Malnourished infants are at increased risk of pneumonia, diarrhea and other infectious diseases contributing to their increased risk of mortality [2], and in the long-term may have permanent cognitive impairment as well as small stature in adulthood [3]. Promoting breastfeeding during infancy has health benefits for mothers and infants and is a cornerstone in infant nutritional programs. However, adherence to recommended breastfeeding practices is low in many sub-Saharan African countries.

Classified by the World Bank as one of the poorest countries in the world, Uganda’s fertility rate is high at 8.7 births per woman [4], but adherence to recommended infant feeding practices is low, contributing the country’s high prevalence of infant malnutrition and low life expectancy.^5^ Overall in Uganda, 98% of mothers initiate breastfeeding after birth, but 27% receive one or more prelacteal feedings and only 42% are exclusively breastfeeding at 4–5 months of age [5]. While 97% had at least one antenatal visit, only 29% had an antenatal visit in the first trimester. Stunting, defined as a height-for-age z-score <-2, is highly prevalent, occurring for 36% of children 24–35 months of age. Developing a better understanding of factors in Uganda contributing to infant feeding practices might therefore have important implications for health. Our study aimed to use data collected in Uganda from focus group discussions (FGDs) and key informant interviews (KIIs) to describe perceptions, experiences and beliefs related to early infant feeding, including barriers and facilitators to optimal breastfeeding practices.

## Methods

In the Kampala area and the Mukono district, in the Central Region of Uganda, we recruited FGD participants using a convenience and snowball sampling method, and conducted 5 focus group discussions (FGDs) with 39 total participants to gather data on experiences, perceptions and beliefs related to nutrition in early infancy from parents whose youngest child had been born in the previous 12 months and from Community Health Workers (CHWs) serving the region. (Table 1). In addition, we conducted 12 key informant interviews (KIIs) with participants including physicians, midwives, and officials from public health and non-governmental organizations (NGOs) (Table 2). All FGDs and KIIs were conducted by trained qualitative researchers who were not health care providers and were fluent in Luganda, the local language. All participants received food during the session and reimbursement for transportation. Participants were asked about specific themes to gather information on local practices and beliefs related to early infant feeding including breastfeeding, breast milk expression and non-breast milk feeding.

**Table 1:** Characteristics of focus groups (FGs) sessions.

Focus Groups’ participants		Area
Group	Number and gender of participants*	
CHWs	10 (F = 9, M = 1)	Mukono HCIV (Mukono district)
CHWs	7 (F = 7, M = 0)	Mukono HCIV (Mukono district)
Mothers with a baby under 1 year	7 (F = 7, M = 0)	Kawala HCIII (Kampala area)
Mothers with a baby under 1 year	8 (F = 8, M = 0)	Mukono HCIV** (Mukono district)
Fathers with a baby under 1 year	7 (F = 0, M = 7)	Mukono HCIV*** (Mukono district)
**TOTAL**	**39 (F = 31, M = 8)**	

* F = Female, M = Male

** Health Centers level IV (HCIV)

*** Health Centers level III (HCIII)

Kampala area was served by the Kawala and Kitebi Health Centers level III and Mukono district was served by the Mukono Health Center level IV

**Table 2:** Key informant interviews (KIIs). Characteristics of semi structured in-depth interviews.

Key informant position/occupation	Key informant affiliation	Date	Length of interview (minutes)
Midwife	Mukono HCIV*	25/09/2019	62 Minutes
Principal Medical Officer	Mukono Municipal Council	27/09/2019	53 Minutes
Midwife Kawala HCIII **	Kawala HCIII	03/10/2019	42 Minutes
Midwife Kitebi HCIII	Kitebi HCIII	03/10/2019	38 Minutes
Proffesor and lecturer	Makerere University, School of Public Health	15/10/2019	42 Minutes
Midwife	Kitebi HCIII	16/10/2019	26 Minutes
Assistant in Charge	Nsambya Hospital, Baby Unit	20/10/2019	39 Minutes
Pediatrician	Nsambya Hospital, Breastmilk Bank	21/10/2019	27 Minutes
Professor and pediatrician	Makerere University	26/10/2019	25 Minutes
Deputy Hospital Director and pediatrician	Mulago Women’s Specialised Hospital	01/11/2019	32 Minutes
WHO official and pediatrician	World Health Organization	15/11/2019	24 Minutes
Pediatrician	Mulago Hospital	26/11/2019	29 Minutes
**TOTAL**			**439 Minutes**

* Health Centers level IV (HCIV)

** Health Centers level III (HCIII)

Each FGD and KII was audio recorded and later transcribed, with translation into English as needed for analysis. Transcripts were analyzed using a process of open coding and thematic analysis based on the themes that emerged from the participants’ narratives. Open coding of transcripts using qualitative analysis software (Atlas.ti) led to a set of multiple codes that captured beliefs, perspectives and nutrition patterns related to early infant feeding. Additional themes emerged during the process through combining multiple codes.

## Results

Major themes identified from the collected data included 1) Infant health concerns; 2) Breastfeeding difficulty; 3) Barriers to breast milk expression; 4) Use of formula and non-formula supplementation; and 5) Poor infant weight gain.

### Infant health concerns

Many infant health concerns came to light during the KIIs, including premature births, low birth weight, domestic violence (leading to premature births and child neglect) and problems in cognitive development and cerebral palsy in babies born prematurely or who suffered asphyxia, as well as diarrhea, cough, fever, gastric infection, bacterial and viral infections, sepsis, anemia, hypernatremia dehydration and obesity.

*“Most babies come with hypernatremic dehydration […]. Then the most common problem, prematurity is too much and conditions with gastric infections are very common we don’t spend a day without admitting someone vomiting.” (Pediatrician Mulago Hospital)*

High HIV prevalence and HIV-related stigma was one of the most frequently cited health concerns. HIV-related stigma was reported to have a strong direct and indirect health impact on all family members, including infants. KIs reported that mothers may refuse to go to prenatal care in government settings for fear of knowing their HIV status and, therefore, give birth through a traditional midwife, exposing the baby to HIV without preventive medication. Moreover, KIs also reported that HIV-related stigma commonly leads many mothers not to disclose their HIV diagnosis to their partners and to not access HIV treatment with subsequent detrimental consequences for the mother, partner and baby’s health.

### Breastfeeding difficulty

Multiple breastfeeding-related issues also emerged during both FGDs and KIIs, including difficulty feeding babies due to extreme poverty, maternal mortality, maternal refusal to breastfeed, breastfeeding with maternal HIV, infant mouth sores described as preventing successful latch and suck and insufficient breast milk due to maternal malnutrition and poor maternal health. Extreme poverty was described as having a direct impact on infant feeding in many different ways. For example, KIIs described that women with HIV are generally abandoned by their partners; these mothers with HIV cannot afford to eat well enough to make sufficient breast milk and are also unable to pay for safe supplementary infant feeding.

*“Then there is poverty too, […] once they are pregnant and the husband gets to know that she is HIV positive in most cases they separate and these mothers cannot afford so they cannot get enough feeds for the young infant.” KII, Midwife*

Extreme poverty and poverty-related occupations, such as street vendors at night, led some mothers to take their children with them while working in extreme conditions, which caused babies to be exposed unhealthy conditions, including low temperatures. In the KIIs, prostitution was also associated with the risk of not breastfeeding and the neglect of children. Poor access to healthcare as well as unsafe food storage due to poor living conditions were also described by KIs as additional barriers to healthy infant nutrition in central Uganda.

Participants in FGDs reported that CHWs informed mothers about the importance of exclusive breastfeeding and that these efforts have been effective at increasing breastfeeding prevalence and duration. Overall, breastfeeding was well regarded by FGD participants. Many believed that breastfeeding contributes to infant health and adequate intellectual development; children who were not breastfed were believed to perform poorly in school. Breastfeeding for a long duration was believed to provide strong immunity and reduce the risk of disease. Moreover, some FGD participants, especially parents, believed that during breastfeeding women cannot conceive and therefore perceived breastfeeding as a beneficial form of family planning. Furthermore, it was reported that breastfeeding contributes to strong bonding between the mother and infant and improves the parents’ relationship.

Despite high levels of knowledge about the potential benefits of breastfeeding, participants describe many barriers preventing breastfeeding. For example, FGD participants reported that HIV positive mothers fear to breastfeed their babies because of risk of HIV transmission. FGD participants also reported that breastfeeding practices were impacted by cultural constraints and that many mothers, especially younger mothers, had concerns about the impact of breastfeeding on maternal physical appearance, such as weight loss and changed breast shape. It was reported that such concerns led some mothers not to breastfeed exclusively or even not breastfeeding at all.

*“[she was] fear of her breasts getting sagged and the baby was given cow milk from day one.” FGD Fathers*

*“Especially the young girls who say am not going to breastfeed, my breasts will be deformed […]. So it is about their breasts, but we have been sensitized about this and women are now trying to breastfeed their babies up to six months.” FGD community health workers*

*“If the mother has been empowered on the importance of breast milk to the baby, she will definitely choose to breastfeed the baby. […] apart from those who want to slay [remain looking attractive] and don’t want their breasts to flap.” FGD Community health workers*

Additionally, FGD participants described that some mothers who produced excessive breast milk believed they developed an unpleasant odor, leading some to take some herbs to reduce milk supply or even to stop breast milk production completely. As emerged during the FGs sessions, men’s extra-marital relations were seen as interrelated with decisions about breastfeeding, with breastfeeding during a period of extra-marital relations thought to be harmful to infant health. Both beneficial and detrimental effects of this belief on breastfeeding duration were reported.

*“I breastfeed a baby up to two years and it is after I have stopped breastfeeding that I have menstrual periods, this helps me too to space my children. […] Some say that if am breastfeeding my relationship with my husband is better, -we are one person-, but the moment I stop breastfeeding the baby, he goes to [local brew place].” FGD community health workers*

*“breastfeeding then it is regarded as a way of keeping their relationship. […] some men too are aware that during the time that the wife is breastfeeding […] [they] should not have extra sexual relationships.” FGD community health workers*

In addition, issues with work-life balance and employment demands were described as limiting breastfeeding options.

*“There are those mothers who find difficulty because they are working for someone. Sometimes even if they are well informed on the benefits of breastfeeding, they can’t do much because their bosses demand that no one goes to work with a baby. They have to express the milk and leave it for the female home helper to give to the baby while the mother goes to work.” FGD mother*

Poverty was one of the main challenges for proper infant nutrition, including breastfeeding. Malnutrition in pregnant and lactating women, especially in single-parent families, were reported to lead both to lack of breast milk and to psychological distress that complicated breastfeeding. Having twins increased the hardship further for women living in poverty. Issues regarding domestic violence and paternal neglect and abandonment of children, as well as barriers to breastfeeding among young prostitutes also emerged during FGDs. As reported FGDs, infants from unwanted pregnancies, young and single mothers, and prostitutes tended not to breastfeed.

*“Maybe the other challenge that makes women so angry/fed-up; you have this baby then your partner starts saying that is not my biological child; you feel so demoralized/demotivated.” FGD mother*

*“Any other reason as to why we are unable to breastfeed for the first six months?*

*yes, they are… there…, sometimes it is due to stress, in most cases if your partner refused you to work […], he refuses you to work yet he has another partner with a breast which is still firm but it ends up falling too. He abandons you, you have no job, you have three children… it is that situation that usually affects us because one has no food. You may endure with the situation but there is a baby you cannot give water to take so it is that stress after delivery that hinders you from breastfeeding.” FGD mother*

*“if you are beating up the woman, you don’t provide her food, do you think she will breastfeed your baby? She will abandon the baby […] but health worker, my request is, you should have intensive sensitization for these youth not to beat up their wives “. FGD father*

*“moderator the other issue is, […] I was beaten by my husband,” Community health worker*

*“The sex workers have a challenge too. They normally say that their clients like licking their nipple and it would be very disgusting for them to find milk there. So they start taking a lot of coffee immediately they give birth so that the breast milk dries and have to put the baby on alternative feeding.” FGD Community health worker*

The impact and extent of the problem of mistreatment and negligent behavior also emerged during the KIIs.

*“In urban here we see of course many babies born to mothers where the care or the fathers abandon or neglect them […] migration young girls come to work in bars […] so someone gets pregnant but the father now leaves the site, relocates, so these babies definitely will have problems”. Pediatrician, KII*

*“And yes […] like violence in homes which also cause prematurity”. Pediatrician, KII*

*“The problem related to newborn mortality today is directly going to maternal health care and mismanagement, so we really have to work a lot in the general public information so that they know when to get pregnant. They can plan a pregnancy at the time when it is wanted because the other social problem we have is abandonment and killed and left in hospitals and so on, on the streets those newborns. Did I mention macerated births and still-births?” Pediatrician, KII*

*“And then treating babies like adults, you know the feeding patterns of babies are not the feeding patterns of the adults, […] the babies feed as much as they want each time the baby wants to feed you have to feed. So some of those patterns are broken because people are working, people are- a mother is selling tomatoes so she abandons the baby because she has to attend to customers and forgets she has to feed the baby so such things”. Midwife, KII*

Additional concerns include breastfeeding in public places, boils on the breast, and inverted and flat nipples.

*“Some mothers either have flat nipples, like the nipple is inside, the baby cannot pull it so you have to pull it out […] And some mothers especially mothers who have delivered premature of course that is physiological torture […]. These Eritreans (speaks softly) they shouldn’t hear me but most of them have issues with inverted nipples flat nipples, so you have to pull them.” KIIS Midwife baby unit*

### Barriers to milk expression

Breast milk expression was reported to be uncommon, although some participants were aware that it was a necessary practice for many premature babies and recommended for working mothers to avoiding early supplementation. Cultural distaste for breast milk expression was reported especially by fathers, who described it as similar to milking a cow or claimed that the practice goes against African culture. However, FGD participants expressed their support for relatives’ breastfeeding after maternal death in childbirth.

*“Honestly, it doesn’t look good, that baby doesn’t listen to her mother’s voice, during breastfeeding […] he misses that contact if he doesn’t breastfeed.” FGD fathers*

*“Honestly, giving expressed milk would not have been a good act because God created this breast milk […] [in addition] whenever you express it, it becomes expired […]” FGD mothers*

*“I have a sibling whose wife had two babies but she passed away […] So she had a sister who had just had a baby, so this baby was given to her […] she breastfed them […]. The way a cow can take milk from another cow and it grows up, that’s why I say that it has no problem … to me.” FGD fathers*

Other concerns expressed by FGD participants about breast milk expression include lack of appropriate hygiene when expressing milk manually or mechanically and when storing breast milk leading to infection and diarrhea. In addition, some participants stated that milk expression can be painful when done manually and that some machine breast milk pumps are “fake” and may injure the nipples. Such difficulties also emerged during the KIIs:

*“[…] the mother who is expressing there, and then you should make sure that the feed is not contaminated and should not give the left overs if she has no fridge, it has to be covered for seven hours […] I mean she has expressed and she has no fridge […] the milk is kept at a normal temperature […] [for] between 7 to 12 hours, It shouldn’t go for so long” KII, Pediatrician*

### Use of formula and non-formula supplementation

A wide range of views regarding infant formula and other nutritional supplements were reported among both parents and CHWs.

*“Most of the mothers I see breastfeed their […] Yet there are those who refuse to breastfeed and make sure that from the time of birth, the baby gets used to taking cow milk.” FGD community health worker*

*“There is a mother who told me that her breast milk is too dilute that the babies cannot get enough milk. This makes the mothers start the babies on porridge early as well as other feeds.” FGD community health worker*

As emerged during the KIIs, affordability is a major issue limiting nutritional choices for infants. As frequently reported, even when necessary or recommended by medical personnel, access to formula is not guaranteed as it is not always available (especially in rural areas) and for many families it is not affordable due to poverty and limited family income.

*“Affordability is an issue because of the costs […] is a challenge… even access, where to buy is a challenge, so you will find that those who use the formula […] are in urban [areas]. KII Pediatrician*

Marketed ready-to-mix formulas were too expensive for many mothers in the study area, and therefore participants reported non-formula supplement usage. A wide range of foods were reported to be used for local non-formula supplements including cow’s milk, glucose water, fruit juice, egg yolk, fish, ghee [clarified butter], groundnuts, soya beans, rice, cassava [yuca] flour and porridges of millet, maize, rice, potatoes, soy and sorghum, among others. Immediately after birth, mothers reported that they are often advised to give glucose in warm water as the mother commences milk production.

*“I was advised by the health worker to give glucose to my first baby since I did not produce breast milk at the time. So I would put the glucose on the tongue, as I waited for breast milk to come[…] finally when milk production begun, I stopped glucose.” FGD mothers*

*“Most of the mothers I see breastfeed their babies while they are still very young but as the babies start growing from 2 months, they are given milk from dairy shops which causes diarrhea to the babies. Yet there are those who refuse to breastfeed and make sure that from the time of birth, the baby gets used to taking cow milk.” FGD community health worker*

*“I have a daughter who had a baby when she was still a child too and resumed school, so I fed that baby on cassava flour with milk. Then for eating I would give groundnuts mixed in pounded silver fish with rice or maize flour I would scoop one spoon of silver fish, one spoonful of groundnuts mix them and put on top of the main food and when it was ready I would feed the baby. The baby grew very well and currently she is in primary two.” FGD community health workers*

*“[…] they supplement with Irish potatoes could be with some milk or porridge*.

*Okay, so apart from the Irish potatoes like our colleague said, the milk, porridge, silver fish, what else is given?*

*Cassava flour mixed in milk even without …*

*And beans too after removing the cover/peeling off the skin.*

*Beans without the cover, someone told us about silver fish mixed with groundnuts and rice*.

*And blue band too*

*You must be innovative/creative*

*And ghee*

*Health worker, we have a woman who had twins, she’s over there, but like we discussed poverty, she would soak soya beans then pound it until it was soft then she would sieve it the next day and it looked like milk then she fed the babies until they were grown. The comment that soya has a bad smell, my friends the other babies, we would pity that woman, its because of the economic condition that made nalongo use soya beans, the next day she would soak it and then sieve it in a cloth, I think it is thick.” FGD Community Health Workers*

*“what do you tell them to feed them on?*

*Silver fish […] Silver fish saves one from so many diseases too*.

*But you haven’t talked about formula*.

*Because we are in a rural area. The people we work with don’t use formula feeds” FGD Community Health Workers*

CHW described giving great attention to instructing mothers on the safe preparation of infant supplements other than formula in order to prevent malnutrition and foodborne intoxication/infections. Mixing cow’s milk or commercially available formula with additional diluted water prior to feeding was reported to be a common practice when commercially available formulas were not affordable.

*“The problem could be maybe if the milk is not well prepared/cooked. […] I have one too who stopped breastfeeding at one week, but we were taught how to prepare cow milk and we did it, and the baby is alive […].” FG fathers*

Some parents believed that expiration dates labels for marketed formulas are removed and fraudulently exchanged for other labels, putting the baby’s health at risk. In addition, some mothers believe formula contains chemicals or does not lead to growth.

*“I really discourage use of supermarket products. My daughter once worked in a supermarket so one time she took long to come back […] Then she told me ‘mother don’t buy anything from any supermarket anymore. We’ve been pulling off papers showing the expiry date replacing new ones. Mum are you listening to me?’ That is the reason as to why we discourage it. You rather pound cassava, silver fish and ground nuts but not giving her tinned food*.

*And pumpkin too is very good, it is very nutritious to the baby*.

*And formula is very expensive too, so this person from the grassroots that we support cannot afford this. I will tell her to rather prepare doodo [vegetables], to prepare for her maize flour and silver fish or beans so that she benefits from the nutrients.” FGD community health workers*

*“Health worker like you said we haven’t talked much about the issue of formula but that has caused so many diseases, sometimes you give thinking that you are giving something good yet you are giving something bad, actually giving her poison” FGD community health workers*.

*“There are things manufactured for babies and an expiry date too is indicated but for some it is not indicated” FGD Mothers*

Fraudulent and adulterated formula practices were also expressed as concerns during the KIIs:

*“The expiry date matters. You cannot take something without knowing its expiry date, it will make babies fall sick, that’s what matters.” KII Midwife*

*“Affordability is a very big issue for a lot of us in Uganda and making sure that you are getting a quality formula milk, you do not know what you are buying and for how long you are buying it.” KIIS Pediatrician*

Additional concerns regarding supplementation of breastfeeding were reported during the FGD sessions and the KIIs. For example, it was believed that when formula was introduced, the number of times the baby is breastfeeding decreases, reducing the production of breast milk.

*“You know one of the things that actually makes mothers’ breast milk reduce is introduction of formula or cow milk and that’s when maybe working women return to work that’s when breast milk flow reduces reason being that the most powerful stimulant of breast milk is baby sucking of the breast […]. So that’s why you know there has been a lot of emphasis on please don’t mix, let the mother exclusively breast feed if she’s to produce more breast milk.” KII pediatrician*

### Infant weight gain

Most parents expressed concern about infant weight and its relationship to health and supplementation. Although most comments focused on underweight children, some concerns about obesity were expressed, while others described beliefs that obesity is a positive sign of proper growth for the baby. These beliefs were also reported to lead to inappropriate nutritional practices.

*“A very small baby is embarrassing, because we have a girl; the other granddaughter to xxx, she had a small baby and would not accept to show her to anyone she would hide her in the house”, FGD mothers*

*“I have our sister who went abroad and left the baby with our mother yet she was weak then the baby was malnourished, they spend the whole day indoors, and she would use her back to sit, the skin was so fragile/soft like this. I told our manager about it then he told me that don’t take the baby to the health facility feed her with living goods products. But it was the feeding so as soon as we fed her the baby was better,” FGD community health workers*

*“So when a baby is born and gains weight at a high speed the mother gets worried too that at this age (month) he is this big, he must be sick. Therefore, I think the baby must be of a medium size because when he loses too much weight, it is bad too.” FG fathers*

*“The baby had very big cheeks. The mother confessed that in most cases she was giving the baby different fatty feeds because she wanted a fat baby. We explained to her that she might see the baby looking good but [she is] not healthy because of the overweight.” KIIS Midwife*

## Discussion

Among parents, community health workers and other stakeholders in Central Uganda, initiation of breastfeeding was reported to be almost universal. However, multiple barriers to ongoing healthy infant nutrition and growth were identified including pain during breastfeeding, preterm birth, nipple problems, return to employment and insufficient milk production, as well as inadequate maternal nutrition and maternal mortality. The use of cow’s milk and diluted cow’s milk for young infants was commonly reported due to lack of affordability of commercially available formula. Additional reported barriers included negative perceptions related to breast milk expression, hygiene and breast milk storage, and concerns regarding appearance and function of breasts which seemed to be prevalent for all and especially highlighted among sex workers.

Our results are consistent with existing literature from both high-income and low- and middle-income countries (LMIC) which has shown that maternal pain, cracked and inverted nipples and insufficient milk production are common barriers to breastfeeding throughout the world [6–8]. Also consistent with existing literature, participants in our study reported concerns about the changed appearance of a mother’s breasts after breastfeeding, and reported avoiding breastfeeding in order to preserve breast appearance [9–12]. However, our findings also highlight the importance of understanding experiences unique to the local context. For example, in contrast with studies conducted in other settings [13–15], our participants in central Uganda did not report that formula marketing influenced maternal breastfeeding decisions; formula was described by participants as not attainable or affordable for the large majority of mothers. Instead, cow’s milk and dilute cow’s milk were the most commonly reported substitutes for breastfeeding, followed by glucose water and other foods and fluids.

Participants in our focus groups also reported strong associations between marital infidelity and breastfeeding practices, with bidirectional causality described. Beliefs that breastfeeding might either prevent or cause infidelity, and that infidelity might impact milk production, appeared to be highly prevalent. To our knowledge, the belief that infidelity might impact milk production has not previously been reported in the scientific literature and is a novel finding from our project, although a qualitative study in northern Malawi [16] found strong local belief that infidelity caused poor infant growth. Local educational interventions specific to this belief might be helpful in promoting healthy infant feeding in central Uganda. Lastly, participants in our study reported commercial sex work as a barrier to breastfeeding. This belief was also reported by a qualitative study in Kenya [17], suggesting that a high prevalence of commercial sex work in the region may be an important contributor to poor infant nutrition. Interventions that reduced the need for new mothers to engage in sex work might greatly improve health conditions for this very vulnerable population of infants.

Our study had several limitations. First, our study enrolled participants in Kampala and Mukono, Uganda, and therefore our results may not be generalizable to other regions of Uganda or to other countries. Second, due to the qualitative nature of our study design, we are unable to report the actual prevalence of breastfeeding or supplementation with other foods or fluids in central Uganda, although our data do suggest that study participants believe these prevalences to be quite high.

Despite these limitations, our results offer strong evidence that mothers in central Uganda are aware of the benefits and importance of exclusive breastfeeding during early infancy but face numerous difficulties in achieving it. Dissemination of information regarding the importance of exclusive breastfeeding appears to have been effective in central Uganda, but unhealthy early feeding practices remain. New interventions are needed that will allow mothers to overcome obstacles to healthy feeding, so that young infants can achieve optimal nutritional outcomes.

## Conclusion

Social, economic and clinical factors present barriers to optimal nutrition during early infancy in Uganda despite widespread awareness that nutrition has a profound influence on infant health. Increasing community knowledge of healthy feeding practices may not be sufficient to achieve current public health goals for infant nutrition. Further research is needed to identify effective methods for achieving these goals in order to improve outcomes during infancy and support population health.
